# Differential roles for pathogenicity islands SPI-13 and SPI-8 in the interaction of *Salmonella* Enteritidis and *Salmonella* Typhi with murine and human macrophages

**DOI:** 10.1186/s40659-017-0109-8

**Published:** 2017-02-15

**Authors:** Rodrigo A. Espinoza, Cecilia A. Silva-Valenzuela, Fernando A. Amaya, Ítalo M. Urrutia, Inés Contreras, Carlos A. Santiviago

**Affiliations:** 1 Laboratorio de Microbiología, Departamento de Bioquímica y Biología Molecular, Facultad de Ciencias Químicas y Farmacéuticas, Universidad de Chile, Santos Dumont 964, Independencia, Santiago Chile; 2 Department of Molecular Biology and Microbiology, Tufts University School of Medicine, Boston, MA 02111 USA

**Keywords:** *Salmonella*, Enteritidis, Typhi, SPI-13, SPI-8, Macrophages, RAW264.7, THP-1

## Abstract

**Background:**

*Salmonella* pathogenicity island (SPI)-13 is conserved in many serovars of *S. enterica*, including *S*. Enteritidis, *S*. Typhimurium and *S*. Gallinarum. However, it is absent in typhoid serovars such as *S*. Typhi and Paratyphi A, which carry SPI-8 at the same genomic location. Because the interaction with macrophages is a critical step in *Salmonella* pathogenicity, in this study we investigated the role played by SPI-13 and SPI-8 in the interaction of *S.* Enteritidis and *S.* Typhi with cultured murine (RAW264.7) and human (THP-1) macrophages.

**Results:**

Our results showed that SPI-13 was required for internalization of *S.* Enteritidis in murine but not human macrophages. On the other hand, SPI-8 was not required for the interaction of *S.* Typhi with human or murine macrophages. Of note, the presence of an intact copy of SPI-13 in a *S*. Typhi mutant carrying a deletion of SPI-8 did not improve its ability to be internalized by, or survive in human or murine macrophages.

**Conclusions:**

Altogether, our results point out to different roles for SPI-13 and SPI-8 during *Salmonella* infection. While SPI-13 contributes to the interaction of *S*. Enteritidis with murine macrophages, SPI-8 is not required in the interaction of *S.* Typhi with murine or human macrophages. We hypothesized that typhoid serovars have lost SPI-13 and maintained SPI-8 to improve their fitness during another phase of human infection.

**Electronic supplementary material:**

The online version of this article (doi:10.1186/s40659-017-0109-8) contains supplementary material, which is available to authorized users.

## Background

The genus *Salmonella* comprises more than 2500 serovars distributed in two species, *S*. *bongori* and *S. enterica* [1, 2]. Some serovars are able to infect a broad range of hosts (generalists), while others only cause disease in a particular host (specialists). *S*. Enteritidis is a generalist serovar that infects mammals and birds causing a typhoid-like infection in susceptible mice and asymptomatic carriage in hens. In humans, it represents a major cause of foodborne gastroenteritis [3, 4]. Conversely, *S*. Typhi is a specialist serovar that infects only humans, causing the systemic disease typhoid fever [5, 6].

**Fig. 1:**
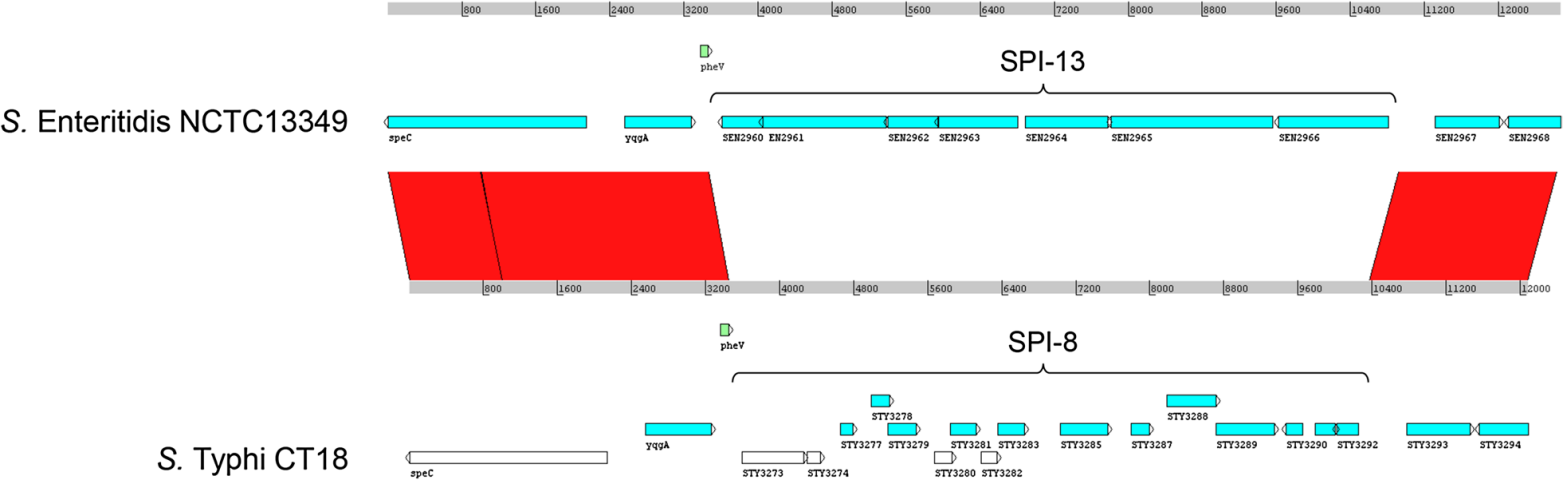
Comparison of the genomic region that includes SPI-13 in *S*. Enteritidis NCTC13349 and SPI-8 in *S*. Typhi CT18. A DNA-based comparison was performed by BLASTN analysis with WebACT (http://www.webact.org/WebACT/home) and visualized with ACT software. Homology between sequences (>98% identity) is highlighted in *red*. *Green boxes* correspond to tRNA-*pheV* genes and *white boxes* correspond to pseudogenes

Most genes required for *Salmonella* pathogenicity are clustered in genomic islands known as *Salmonella* pathogenicity islands (SPIs). So far, 23 SPIs have been described and characterized [7–11]. Five SPIs (SPI-1 to SPI-5) are common to all serovars of *S. enterica*, while the rest is distributed among different serovars and/or strains [7–11]. One such example is SPI-13, which is present in many *S*. *enterica* serovars including *S*. Enteritidis, *S*. Typhimurium, *S*. Cholerasuis and *S*. Gallinarum, but it is absent in *S*. Typhi and *S*. Paratyphi A, which carry SPI-8 in the same genomic location, adjacent to the tRNA-*pheV* gene [8, 9, 12] (Fig. 1). The number of genes within SPI-13 is not clearly defined; different authors have reported that it contains 4 [8], 18 [13] or 22 ORFs [9]. In this study we defined SPI-13 as the region including genes *SEN2960* to *SEN2966* of *S*. Enteritidis because they are flanked by nearly perfect direct repeats. In addition, these are the genes replaced in *S*. Typhi by SPI-8, which includes genes *STY3273* to *STY3292* [14].

It has been reported that genes in SPI-13 from *S.* Typhimurium [15–17] and *S*. Enteritidis [18] are expressed within macrophages in vitro, and are required for systemic infection in mice [9, 19–22]. These genes are homologues to the *ripABC* operon of *Yersinia pestis*, which encodes proteins required for the degradation of itaconate produced by macrophages during infection [23].

In comparison to SPI-13, much less information is available on SPI-8. In *S*. Typhi, this pathogenicity island contains a degenerate integrase gene, two bacteriocin pseudogenes and intact genes encoding proteins conferring immunity to these bacteriocins [14]. In addition, *STY3280* has been shown to be expressed within human macrophages [24].

In this study, we investigated the roles of SPI-13 and SPI-8 in the interaction of *S.* Enteritidis and *S.* Typhi with cultured murine and human macrophages. Previously, other authors have demonstrated that both serovars are internalized by murine macrophages; but only *S.* Enteritidis is able to survive intracellularly [25]. In contrast, *S*. Enteritidis has a low ability to survive intracellularly in human macrophages, whereas *S*. Typhi survives and proliferates for long periods, showing less cytotoxicity than other serovars [25]. We hypothesized that the differential distribution of SPI-13 and SPI-8 in these two serovars might drive, in part, the different phenotypes observed in vitro in macrophages of different origins.

## Methods

### Bacterial strains, media, and growth conditions

Bacterial strains used in this study are listed in Table 1. All *S*. Enteritidis and *S*. Typhi strains are derivatives of wild-type strains NCTC13349 [13] and STH2370 [26], respectively. Bacteria were routinely grown in Luria–Bertani (LB) medium (10 g/L tryptone, 5 g/L yeast extract, 5 g/L NaCl) at 37 °C with agitation. When required, LB medium was supplemented with ampicillin (Amp; 100 mg/L) or kanamycin (Kan; 50 mg/L). Media were solidified by the addition of agar (15 g/L).

**Table 1:** Bacterial strains used in the present study

Strain	Phenotype/genotype	Source or reference
*Salmonella*		
*S*. Enteritidis NCTC13349	Wild-type strain	[13]
*S*. Enteritidis ∆SPI-13	NCTC13349 ∆(*SEN2960*-*SEN2966*)::FRT	This study
*S*. Enteritidis ∆SPI-13/pSPI-13	NCTC13349 ∆(*SEN2960*-*SEN2966*)::FRT/pBAD-TOPO::(*SEN2960*-*SEN2966*)	This study
*S*. Typhi STH2370	Wild-type strain	[26]
*S*. Typhi ∆SPI-8	STH2370 ∆(*STY3273*-*STY3292*)::FRT	This study
*S*. Typhi ∆SPI-8/pSPI-8	STH2370 ∆(*STY3273*-*STY3292*)::FRT/pBAD-TOPO::(*STY3273*-*STY3292*)	This study
*S*. Typhi ∆SPI-8/pSPI-13	STH2370 ∆(*STY3273*-*STY3292*)::FRT/pBAD-TOPO::(*SEN2960*-*SEN2966)*	This study
*Escherichia coli*		
TOP10	K12 F^−^ Ф80*lacZ*∆M15 ∆(*lacZYA*-*argF*)U169 *deoR recA1 endA1 hsdR17*(*r*_k_^−^, *m*_k_^+^) *phoA supE44 thi*–*1 gyrA96 relA1* λ^−^	Invitrogen

### Construction of mutant strains

All mutant strains were constructed by the Red-swap method [27] with modifications [21], using plasmid pCLF4 (Kan^R^, GenBank accession number EU629214) as template and specific primers listed in Additional file 1: Table S1. The Kan-resistance cassette introduced by this procedure was eliminated by recombination using the FLP helper plasmid pCP20 [27, 28]. Each mutation was confirmed by PCR amplification using primers flanking the sites of substitution (Additional file 1: Table S1).

### Construction of plasmids

SPI-13 and SPI-8 were amplified from strains NCTC13349 and STH2370, respectively, using *PfuUltra II* high-fidelity DNA polymerase (Stratagene) and specific primers listed in Additional file 1: Table S1. Each PCR product was purified from 1% agarose gels using the “QIAquick Gel Extraction Kit” (QIAGEN). Purified PCR products were adenylated at the 3′ end and cloned into pBAD-TOPO using the “pBAD-TOPO TA Expression Kit” (Invitrogen). Recombinant plasmids pSPI-13 and pSPI-8 were transformed into competent *E. coli* TOP10 (Invitrogen). The presence and orientation of the insert in each plasmid was confirmed by PCR amplification using primers listed in Additional file 1: Table S1. Finally, different strains of *S*. Enteritidis and *S*. Typhi were transformed by electroporation with these plasmids for complementation assays.

### Cell lines and culture conditions

RAW264.7 murine macrophages were grown as monolayers in Dulbecco’s modified Eagle medium (DMEM) supplemented with 10% fetal bovine serum (FBS). THP-1 human monocytes were grown in suspension using RPMI-1640 medium supplemented with 2 mM l-glutamine, 10% FBS and 50 μM β-mercaptoethanol. Both cell lines were maintained in an air-jacketed incubator (NuAire) at 37 °C in the presence of 5% CO_2_.

### Bacterial infection assays

For infection assays using RAW264.7 macrophages, monolayers were prepared by seeding ~2.5 × 10^5^ cells/well in 48-well plates (Nunc) and incubating for 20 h at 37 °C in a 5% CO_2_ atmosphere. From each bacterial culture grown overnight in LB at 37 °C with agitation, a 1/100 dilution in 5 mL of LB was prepared, and grown under the same conditions until reaching an OD_600_ = 0.4–0.6. Then, bacteria were centrifuged and washed with phosphate buffered saline (PBS), suspended in 100 μL of DMEM and added to the macrophages at a multiplicity of infection (MOI) of ~50 bacteria/cell. After 45 min of incubation at 37 °C, macrophages were washed three times with PBS containing 200 mg/L gentamicin and further incubated for 1 h in DMEM containing 200 mg/L gentamicin to kill extracellular bacteria. Then, cells from three wells were washed with PBS, and lysed with 0.5% sodium deoxycholate. Internalized bacteria were enumerated by serial dilution and plating on LB agar. The percentage of internalization was calculated as follows: 100 × (CFU_internalized_/CFU_inoculum_). In the remaining six wells, medium was replaced by DMEM containing 20 mg/L gentamicin and cells were incubated for 2 h (three wells) or 20 h (three wells). Then, cells were washed, lysed and CFU were determined as described. The percentage of intracellular survival was calculated as follows: 100 × (CFU_2 or 20h_/CFU_internalized_).

For infection assays using THP-1 monocytes, ~5 × 10^5^ cells were seeded in 1.7 mL tubes and incubated for 20 h at 37 °C in a 5% CO_2_ atmosphere. Bacteria were grown as indicated above, washed with PBS and suspended in 100 μL of RPMI-FBS without β-mercaptoethanol. THP-1 cells were spun down and the bacterial suspension was added, resulting in a MOI of ~50 bacteria/cell. Internalization and intracellular survival were determined as described above.

### Cytotoxicity assays

To determine the cytotoxicity produced by *Salmonella* strains during macrophage infection, lactate dehydrogenase (LDH) release was measured using “CytoTox96 Non-Radioactive Cytotoxicity Assay” (Promega), as recommended by the manufacturer.

### Statistical analysis

Statistical significance of differences in the data was determined via one-way ANOVA with Dunnett’s test using the GraphPad Prism v6.0 software.

## Results

### SPI-13 is required for *S.* Enteritidis internalization in murine macrophages

**Fig. 2:**
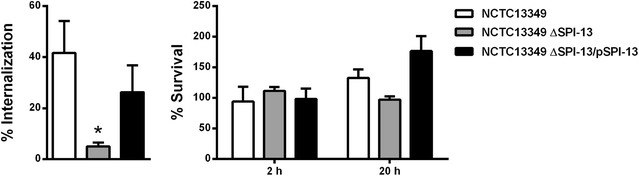
Contribution of SPI-13 to the internalization and intracellular survival of *S*. Enteritidis in murine macrophages. Infection assays were conducted as described in “Methods” section to determine the internalization and intracellular survival of *S*. Enteritidis strains in RAW264.7 macrophages. Graphics show mean values of at least 3 independent assays ± SEM. Statistical significance was determined using a one-way ANOVA with Dunnett’s test (*P < 0.05)

It has been shown that several SPI-13 genes contribute to *Salmonella* pathogenesis in mice and chickens [9, 12, 19–22]. Because survival within macrophages is a crucial step in *Salmonella* systemic infection, we evaluated the contribution of SPI-13 in the interaction of *S*. Enteritidis with RAW264.7 macrophages. To this end, we tested the ability of the wild-type strain NCTC13349, an isogenic ∆SPI-13 mutant and the mutant complemented in *trans* with an intact copy of SPI-13, to be internalized by and survive within RAW264.7 macrophages. Our results showed that deletion of SPI-13 significantly impaired *S.* Enteritidis internalization in comparison to the wild-type strain. This phenotype was reverted by the introduction of a plasmid carrying an intact copy of SPI-13 (Fig. 2). On the other hand, deletion of SPI-13 did not affect *S.* Enteritidis survival after 2 or 20 h of infection (Fig. 2). The presence of the empty vector did not alter the phenotypes observed for the wild-type strain or the ∆SPI-13 mutant in our infection assays (data not shown). In addition, all strains showed similar cytotoxicity to RAW264.7 macrophages, as evaluated by LDH release during infections (data not shown).

### SPI-8 is not required for *S.* Typhi internalization or intracellular survival in murine macrophages

**Fig. 3:**
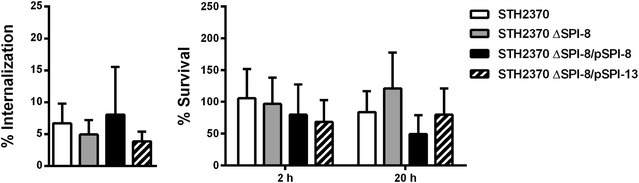
Contribution of SPI-8 and SPI-13 to the internalization and intracellular survival of *S.* Typhi in murine macrophages. Infection assays were conducted as described in “Methods” section to determine the internalization and intracellular survival of *S*. Typhi strains in RAW264.7 macrophages. Graphics show mean values of at least 3 independent assays ± SEM. Statistical significance was determined using a one-way ANOVA with Dunnett’s test

To determine the role played by SPI-8 in the interaction of *S*. Typhi with murine macrophages, we tested the ability of the wild-type strain STH2370, an isogenic ∆SPI-8 mutant and the mutant complemented in *trans* with an intact copy of SPI-8, to be internalized by and survive within RAW264.7 cells. No significant differences compared to the wild type were found when the ∆SPI-8 mutant and the complemented strain were analyzed (Fig. 3). In addition, the presence/absence status of SPI-8 did not affect the cytotoxicity of *S*. Typhi on RAW264.7 macrophages (data not shown).

In view of our results with *S*. Enteritidis (Fig. 2), we tested if the presence of SPI-13 could improve the internalization or survival of *S.* Typhi ∆SPI-8 within murine macrophages. To do this, we performed infection assays using the *S.* Typhi ∆SPI-8 mutant carrying an intact copy of SPI-13. Our results showed that the presence of SPI-13 did not increase the internalization or intracellular survival of *S*. Typhi ∆SPI-8 in RAW264.7 macrophages (Fig. 3). Furthermore, the presence of this island did not affect the cytotoxicity of *S.* Typhi ∆SPI-8 on RAW264.7 macrophages (data not shown).

### Neither SPI-8 nor SPI-13 contribute to the internalization or intracellular survival of *S*. Typhi and *S.* Enteritidis in human macrophages

**Fig. 4:**
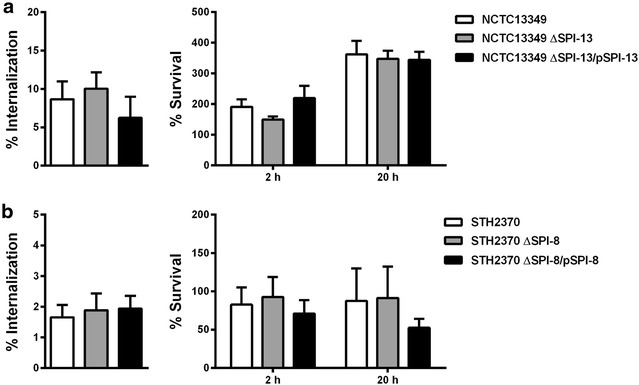
Contribution of SPI-13 and SPI-8 to the internalization and intracellular survival of *S.* Enteritidis and *S.* Typhi in human macrophages. Infection assays were conducted as described in “Methods” section to determine the internalization and intracellular survival of *S.* Enteritidis (**a**) and *S*. Typhi (**b**) strains in THP-1 macrophages. Graphics show mean values of at least 3 independent assays ± SEM. Statistical significance was determined using a one-way ANOVA with Dunnett’s test

Next, we aimed to determine whether SPI-13 and SPI-8 contribute to the internalization or intracellular survival of *S.* Enteritidis and *S*. Typhi, respectively, in human macrophages. To do this, we first performed infection assays in THP-1 human macrophages using wild-type *S.* Enteritidis, the ∆SPI-13 mutant, and the mutant complemented in *trans* with an intact copy of SPI-13. We did not observe differences in internalization, intracellular survival (Fig. 4a) and cytotoxicity (data not shown) between the wild-type strain and the mutants tested. Similar results were obtained when THP-1 macrophages were infected with wild-type *S.* Typhi, the ∆SPI-8 mutant, and the mutant complemented in *trans* with an intact copy of SPI-8 (Fig. 4b).

## Discussion

Although SPI-13 was identified in *S*. Gallinarum [12], it has been described in several members of *S*. *enterica* [8, 9, 13, 29]. The number of genes conforming SPI-13 is not clearly defined. In fact, different authors have reported that this island contains 4 [8], 18 [13] or 22 genes [9]. In this study, we defined SPI-13 as the region encompassing genes *SEN2960* to *SEN2966* in *S*. Enteritidis because these genes are adjacent to the tRNA-*pheV* gene and are flanked by nearly perfect direct repeats. Of note, these 7 genes are replaced in the genome of typhoid *Salmonella* serovars such as *S*. Typhi and *S*. Paratyphi A by genes composing SPI-8 [8, 9, 14]. We investigated whether this genetic difference contributes to the differential intracellular survival reported for *S*. Enteritidis and *S*. Typhi in macrophages of murine and human origin [25].

Our results indicate that SPI-13 is required for internalization of *S*. Enteritidis in RAW264.7 macrophages. In agreement with this observation, a null mutant of gene *SEN2961* also presented an internalization defect in HD-11 chicken macrophages [18]. Reduced internalization by macrophages can affect *S*. Enteritidis pathogenicity because bacterial uptake by phagocytes is required for systemic spread of the pathogen and, as mentioned, SPI-13 is required for systemic colonization of mice and chicken by different *Salmonella* serovars [9, 12, 19–22].

Our data also indicate that SPI-13 is not involved in the intracellular survival of *S*. Enteritidis in RAW264.7 macrophages. These results do not agree with a recent study showing that deletion of SPI-13 (genes *SEN2960* to *SEN2977*) resulted in increased uptake and impaired survival of *S*. Enteritidis in RAW264.7 macrophages [20]. The discrepancies between results from both studies may be due to differences in the *S.* Enteritidis strain employed and the number of SPI-13 genes deleted (7 vs. 18). Regarding this last point, the function of *S*. Enteritidis genes in the *SEN2967*-*SEN2977* region (not included in our analysis) has been recently predicted and some of them may be required for intracellular survival in macrophages. For instance, *SEN2973* encodes the antiadaptor protein IraD, which in *S*. Typhimurium has been reported to increase the stability of the sigma stress factor RpoS during oxidative stress [30]. In addition, transposon insertion mutants of *S*. Enteritidis in genes *SEN2972*, *SEN2976* and *SEN2977* present systemic colonization defects in mice [22]. Furthermore, the ∆SPI-13 mutant used by the authors of the other study kept the kanamycin-resistance cassette replacing the island, which may generate polar effects on genes surrounding the site of mutation.

Remarkably, genes *SEN2961*-*SEN2963* of *S*. Enteritidis are homologous to the *ripABC* operon of *Y. pestis*. This operon was originally described as required for *Y. pestis* survival and replication within activated macrophages [31–33]. More recently, it was reported that *Y. pestis ripABC* operon encodes proteins with itaconate Co-A transferase, itaconil Co-A hydratase and citralil Co-A lyase activities, which together degrade itaconate and promote pathogenicity [23]. Itaconate is produced by macrophages in response to bacterial infection. This compound is a potent inhibitor of isocitrate lyase that blocks the glyoxylate cycle used by bacteria such as *Salmonella* and *Mycobacterium* to survive intracellularly [23, 34].

In contrast to our observations in RAW264.7 murine macrophages, SPI-13 was not required for internalization or survival of *S*. Enteritidis in THP-1 human macrophages. A possible explanation for these observations is that human macrophages produce itaconate at lower levels compared with mouse macrophages [34]. Hence, enzymes for itaconate degradation encoded in SPI-13 would not be needed in these macrophages. This hypothesis is supported by the fact that the genome of human pathogens *S*. Typhi and *S*. Paratyphi A do not contain SPI-13. Furthermore, our results showed that SPI-8 was not required for internalization or intracellular survival of *S*. Typhi in RAW264.7 murine macrophages or THP-1 human macrophages. We hypothesize that SPI-8 has been maintained in the genome of typhoid *Salmonella* serovars because it encodes factors required during other steps of human infection. For instance, proteins encoded in SPI-8 conferring immunity to bacteriocins [14] could improve bacterial fitness of typhoid serovars in the human gut. Further studies are required to confirm this hypothesis.

## Conclusions

In this study we evaluated the contribution of pathogenicity islands SPI-13 and SPI-8 in the interaction of *S*. Enteritidis and *S*. Typhi with cultured murine (RAW264.7) and human (THP-1) macrophages. SPI-13 is present in the genome of many serovars of *Salmonella enterica*, including *S*. Enteritidis. However, this pathogenicity island is absent in the genome of typhoid serovars such as *S*. Typhi and *S*. Paratyphi C, which carry SPI-8 in the same genomic location. Our results indicate that SPI-13 is required for internalization of *S.* Enteritidis in murine but not in human macrophages. On the other hand, SPI-8 is not required for the internalization or survival of *S.* Typhi in human or murine macrophages. Thus, our results indicate different roles for SPI-13 and SPI-8 during *Salmonella* infection.

## Additional file

**Additional file 1: Table S1.** Primers used in the present study.

## References

[1] Brenner FW, Villar RG, Angulo FJ, Tauxe R, Swaminathan B (2000). *Salmonella* nomenclature. J Clin Microbiol.

[2] Reeves MW, Evins GM, Heiba AA, Plikaytis BD, Farmer JJ (1989). Clonal nature of *Salmonella typhi* and its genetic relatedness to other Salmonellae as shown by multilocus enzyme electrophoresis, and proposal of *Salmonella bongori* comb. nov. J Clin Microbiol.

[3] Guard-Petter J (2001). The chicken, the egg and *Salmonella enteritidis*. Environ Microbiol.

[4] Patrick ME, Adcock PM, Gomez TM, Altekruse SF, Holland BH, Tauxe RV, Swerdlow DL (2004). *Salmonella* Enteritidis infections, United States, 1985–1999. Emerg Infect Dis.

[5] Crump JA, Luby SP, Mintz ED (2004). The global burden of typhoid fever. Bull World Health Organ.

[6] House D, Bishop A, Parry C, Dougan G, Wain J (2001). Typhoid fever: pathogenesis and disease. Curr Opin Infect Dis.

[7] Blondel CJ, Jimenez JC, Contreras I, Santiviago CA (2009). Comparative genomic analysis uncovers 3 novel loci encoding type six secretion systems differentially distributed in *Salmonella* serotypes. BMC Genom.

[8] Desai PT, Porwollik S, Long F, Cheng P, Wollam A, Bhonagiri-Palsikar V, Hallsworth-Pepin K, Clifton SW, Weinstock GM, McClelland M (2013). Evolutionary genomics of *Salmonella enterica* subspecies. mBio.

[9] Haneda T, Ishii Y, Danbara H, Okada N (2009). Genome-wide identification of novel genomic islands that contribute to *Salmonella* virulence in mouse systemic infection. FEMS Microbiol Lett.

[10] Hayward MR, Jansen V, Woodward MJ (2013). Comparative genomics of *Salmonella enterica* serovars Derby and Mbandaka, two prevalent serovars associated with different livestock species in the UK. BMC Genom.

[11] Hensel M (2004). Evolution of pathogenicity islands of *Salmonella enterica*. Int J Med Microbiol.

[12] Shah DH, Lee MJ, Park JH, Lee JH, Eo SK, Kwon JT, Chae JS (2005). Identification of *Salmonella gallinarum* virulence genes in a chicken infection model using PCR-based signature-tagged mutagenesis. Microbiology.

[13] Thomson NR, Clayton DJ, Windhorst D, Vernikos G, Davidson S, Churcher C, Quail MA, Stevens M, Jones MA, Watson M (2008). Comparative genome analysis of *Salmonella* Enteritidis PT4 and *Salmonella* Gallinarum 287/91 provides insights into evolutionary and host adaptation pathways. Genome Res.

[14] Parkhill J, Dougan G, James KD, Thomson NR, Pickard D, Wain J, Churcher C, Mungall KL, Bentley SD, Holden MT (2001). Complete genome sequence of a multiple drug resistant *Salmonella enterica* serovar Typhi CT18. Nature.

[15] Eriksson S, Lucchini S, Thompson A, Rhen M, Hinton JC (2003). Unravelling the biology of macrophage infection by gene expression profiling of intracellular *Salmonella enterica*. Mol Microbiol.

[16] Shi L, Adkins JN, Coleman JR, Schepmoes AA, Dohnkova A, Mottaz HM, Norbeck AD, Purvine SO, Manes NP, Smallwood HS (2006). Proteomic analysis of *Salmonella enterica* serovar Typhimurium isolated from RAW 264.7 macrophages: identification of a novel protein that contributes to the replication of serovar Typhimurium inside macrophages. J Biol Chem.

[17] Srikumar S, Kroger C, Hebrard M, Colgan A, Owen SV, Sivasankaran SK, Cameron AD, Hokamp K, Hinton JC (2015). RNA-seq brings new insights to the intra-macrophage transcriptome of *Salmonella* Typhimurium. PLoS Pathog.

[18] Zhao Y, Jansen R, Gaastra W, Arkesteijn G, van der Zeijst BA, van Putten JP (2002). Identification of genes affecting *Salmonella enterica* serovar enteritidis infection of chicken macrophages. Infect Immun.

[19] Chakraborty S, Chaudhuri D, Balakrishnan A, Chakravortty D (2014). *Salmonella* methylglyoxal detoxification by STM3117-encoded lactoylglutathione lyase affects virulence in coordination with *Salmonella* pathogenicity island 2 and phagosomal acidification. Microbiology.

[20] Elder JR, Chiok KL, Paul NC, Haldorson G, Guard J, Shah DH (2016). The *Salmonella* pathogenicity island 13 contributes to pathogenesis in streptomycin pre-treated mice but not in day-old chickens. Gut Pathog.

[21] Santiviago CA, Reynolds MM, Porwollik S, Choi SH, Long F, Andrews-Polymenis HL, McClelland M (2009). Analysis of pools of targeted *Salmonella* deletion mutants identifies novel genes affecting fitness during competitive infection in mice. PLoS Pathog.

[22] Silva CA, Blondel CJ, Quezada CP, Porwollik S, Andrews-Polymenis HL, Toro CS, Zaldivar M, Contreras I, McClelland M, Santiviago CA (2012). Infection of mice by *Salmonella enterica* serovar Enteritidis involves additional genes that are absent in the genome of serovar Typhimurium. Infect Immun.

[23] Sasikaran J, Ziemski M, Zadora PK, Fleig A, Berg IA (2014). Bacterial itaconate degradation promotes pathogenicity. Nat Chem Biol.

[24] Faucher SP, Curtiss R, Daigle F (2005). Selective capture of *Salmonella enterica* serovar Typhi genes expressed in macrophages that are absent from the *Salmonella enterica* serovar Typhimurium genome. Infect Immun.

[25] Schwan WR, Huang XZ, Hu L, Kopecko DJ (2000). Differential bacterial survival, replication, and apoptosis-inducing ability of *Salmonella* serovars within human and murine macrophages. Infect Immun.

[26] Valenzuela C, Ugalde JA, Mora GC, Alvarez S, Contreras I, Santiviago CA (2014). Draft genome sequence of *Salmonella enterica* serovar Typhi strain STH2370. Genome Announc.

[27] Datsenko KA, Wanner BL (2000). One-step inactivation of chromosomal genes in *Escherichia coli* K-12 using PCR products. Proc Natl Acad Sci USA.

[28] Cherepanov PP, Wackernagel W (1995). Gene disruption in *Escherichia coli*: Tc^R^ and Km^R^ cassettes with the option of Flp-catalyzed excision of the antibiotic-resistance determinant. Gene.

[29] McClelland M, Sanderson KE, Spieth J, Clifton SW, Latreille P, Courtney L, Porwollik S, Ali J, Dante M, Du F (2001). Complete genome sequence of *Salmonella enterica* serovar Typhimurium LT2. Nature.

[30] Battesti A, Tsegaye YM, Packer DG, Majdalani N, Gottesman S (2012). H-NS regulation of IraD and IraM antiadaptors for control of RpoS degradation. J Bacteriol.

[31] Pujol C, Grabenstein JP, Perry RD, Bliska JB (2005). Replication of *Yersinia pestis* in interferon gamma-activated macrophages requires *ripA*, a gene encoded in the pigmentation locus. Proc Natl Acad Sci USA.

[32] Torres R, Chim N, Sankaran B, Pujol C, Bliska JB, Goulding CW (2012). Structural insights into RipC, a putative citrate lyase beta subunit from a *Yersinia pestis* virulence operon. Acta Crystallogr Sect F Struct Biol Cryst Commun.

[33] Torres R, Swift RV, Chim N, Wheatley N, Lan B, Atwood BR, Pujol C, Sankaran B, Bliska JB, Amaro RE (2011). Biochemical, structural and molecular dynamics analyses of the potential virulence factor RipA from *Yersinia pestis*. PLoS ONE.

[34] Michelucci A, Cordes T, Ghelfi J, Pailot A, Reiling N, Goldmann O, Binz T, Wegner A, Tallam A, Rausell A (2013). Immune-responsive gene 1 protein links metabolism to immunity by catalyzing itaconic acid production. Proc Natl Acad Sci USA.

