# Supplementary material for: Differential roles for pathogenicity islands SPI-13 and SPI-8 in the interaction of Salmonella Enteritidis and Salmonella Typhi with murine and human macrophages
Source: Biol Res. 2017 Feb 15;50:5. doi: 10.1186/s40659-017-0109-8 (PMC5311848; doi:10.1186/s40659-017-0109-8)
Supplement: Supplementary file 1 — Additional file 1: Table S1. Primers used in the present study. [file 40659_2017_109_MOESM1_ESM.doc]

Table S1.- Primers used in the present study.

| **Primer** | **Sequence** |
| --- | --- |
| **Mutant constructions** | |
| STM3123_(H1+P1) | GGACAGAATAATGTTGAATCTCAACACATTACGGCAACAAGTGCAGGCTGGAGCTGCTTC |
| STM3117_(H2+P2) | AGTTTTAACATTAAACATACTGCGAAATTTCAATTAAGTTCATATGAATATCCTCCTTAG |
| STM3117_Out5 | ACAAGGCGTCGTTACCAATC |
| STM3123_Out3 | GGACGTTCGTTTTGCGTACT |
| SPI-8_(H1+P1) | TTGGTGGTCAACTCGGTGCTCAGTTCGGTTAGATAAAGGAGTGCAGGCTGGAGCTGCTTC |
| SPI-8_(H2+P2) | GGGTAACCTGTTGATTTTATAAATAAAAACGGACGCCATACATATGAATATCCTCCTTAG |
| SPI-8_Junct-R | TGCGTTATTTAAGTGGTTCATGCCG |
| SPI-8_Junct-L | GAGTTTCAGGCAGCCGGTA |
| K1 | CAGTCATAGCCGAATAGCCT |
| **Cloning of SPI-13 and SPI-8** | |
| SPI-13_clone_Out5+BamHI | GC**GGATCC**TCCATTAGACCAATATAAACGGATGCG |
| SPI-13_clone_Out3+BamHI | GC**GGATCC**GGGCGCAGTAAAATAGTGGTATGTTGA |
| SPI-8_clone_Out5+BamHI | GC**GGATCC**TGGAAAAACTCTTCCCAGCGAATTT |
| SPI-8_clone_Out3+BamHI | GC**GGATCC**AAAGCGGTGGCTGCCAGTATATTTT |
| Amp-R_Forward | CTGAAGATCAGTTGGGTGCA |
| Amp-R_Reverse | AAGTTGGCCGCAGTGTTATC |

Underlined sequences indicate regions that anneal to the 5’ or 3’ end of the antibiotic-resistance cassette in template vector pCLF4 (GenBank accession number EU629214). Sequences in bold indicate *Bam*HI restriction sites.
